# The Role of Slr0151, a Tetratricopeptide Repeat Protein from *Synechocystis* sp. PCC 6803, during Photosystem II Assembly and Repair

**DOI:** 10.3389/fpls.2016.00605

**Published:** 2016-05-03

**Authors:** Anna Rast, Birgit Rengstl, Steffen Heinz, Andreas Klingl, Jörg Nickelsen

**Affiliations:** ^1^Molekularbiologie der Pflanzen, Biozentrum der Ludwig-Maximilians-Universität MünchenPlanegg-Martinsried, Germany; ^2^Pflanzliche Entwicklungsbiologie, Biozentrum der Ludwig-Maximilians-Universität MünchenPlanegg-Martinsried, Germany

**Keywords:** *Synechocystis*, biogenesis center, TPR protein, photosystem II, thylakoid membrane

## Abstract

The assembly and repair of photosystem II (PSII) is facilitated by a variety of assembly factors. Among those, the tetratricopeptide repeat (TPR) protein Slr0151 from *Synechocystis* sp. PCC 6803 (hereafter *Synechocystis*) has previously been assigned a repair function under high light conditions (Yang et al., 2014). Here, we show that inactivation of *slr0151* affects thylakoid membrane ultrastructure even under normal light conditions. Moreover, the level and localization of Slr0151 are affected in a variety of PSII-related mutants. In particular, the data suggest a close functional relationship between Slr0151 and Sll0933, which interacts with Ycf48 during PSII assembly and is homologous to PAM68 in *Arabidopsis thaliana*. Immunofluorescence analysis revealed a punctate distribution of Slr0151 within several different membrane types in *Synechocystis* cells.

## Introduction

The ability of plastid-bearing organisms to perform oxygenic photosynthesis was inherited from an ancient cyanobacterium about 2.4 billion years ago. In present-day cyanobacteria, the photosynthetic electron transport chain (PET) is embedded in an internal membrane system made up of thylakoids (Hohmann-Marriott and Blankenship, 2011). The PET is fueled by electrons originating from the water-splitting complex within PSII, which is therefore considered to be the heart of photosynthesis. Recently, the structural analysis of PSII has revealed detailed insights into the architecture and working mode of its Mn_4_CaO_5_ cluster, where water is oxidized and molecular oxygen is released (Umena et al., 2011; Kupitz et al., 2014; Suga et al., 2015).

Overall, PSII comprises at least 20 protein subunits as well as numerous organic and inorganic co-factors. All these components have to be assembled in a strictly coordinated manner in both time and space. The emerging picture indicates that the assembly process is initiated at specialized, biogenic thylakoid membrane (TM) regions and proceeds step-wise until the active PSII super-complex is formed as part of photosynthetically active thylakoids (Komenda et al., 2012; Nickelsen and Rengstl, 2013; Nickelsen and Zerges, 2013; Rast et al., 2015).

In the cyanobacterium *Synechocystis* sp. PCC 6803 (hereafter *Synechocystis*), the initial steps in *de novo* PSII assembly have been proposed to take place at biogenesis centers (BC) where the thylakoids converge on the plasma membrane (PM; van de Meene et al., 2006; Schottkowski et al., 2009a; Stengel et al., 2012; Nickelsen and Rengstl, 2013). The precise architecture of BCs is not yet fully understood; however, they are characterized by the accumulation of the PSII assembly factor PratA, which delivers Mn to the precursor of the D1 reaction-center protein (pD1; Stengel et al., 2012). The C-terminal extension of pD1 is then processed by the protease CtpA (Anbudurai et al., 1994; Zak et al., 2001; Komenda et al., 2007). Concomitantly, the first detectable PSII assembly intermediate, i.e., the reaction-center complex (RC), is formed by the attachment of the D2-Cyt *b*_559_ module which is aided by the assembly factor Ycf48, a homolog of Hcf136 from *Arabidopsis thaliana* (Komenda et al., 2004, 2008). Via the interaction of Ycf48 with the PAM68-homolog Sll0933, the inner core antenna proteins CP47 and CP43 bind successively to the RC complex, forming a PSII monomer that still lacks the lumenal subunits of the oxygen-evolving complex (OEC; Komenda et al., 2004; Rengstl et al., 2013). Finally, the OEC is built with the help of the assembly factors CyanoP and Psb27, yielding a fully functional PSII monomer (Nowaczyk et al., 2006; Becker et al., 2011; Komenda et al., 2012; Cormann et al., 2014).

Moreover, due to its susceptibility to photodamage, PSII needs to be repaired about every 30 min (Prasil et al., 1992; Mulo et al., 1998). In this process, PSII is disassembled by removing the PsbO, PsbV, PsbU, and CyanoQ subunits, followed by CP43 (Mulo et al., 2012; Mabbitt et al., 2014). Damaged D1 protein is then degraded by the FtsH2/H3 protease complex (Silva et al., 2003; Komenda et al., 2006; Boehm et al., 2012; Mabbitt et al., 2014) and replaced by newly synthesized D1, which is co-translationally inserted into the complex. Next, CP43 re-attaches and functional PSII is restored (Zhang et al., 1999; Komenda et al., 2008; Mulo et al., 2012; Mabbitt et al., 2014).

As outlined above, recent years have seen the discovery of many accessory factors that are involved in catalyzing distinct PSII assembly/repair steps. Many of these have been found to belong to the so-called family of TPR (tetratricopeptide repeat) proteins (Heinz et al., 2016; Rast et al., 2015). TPR proteins represent solenoid-like, “scaffold” proteins which are distributed throughout all kingdoms of life (for a recent review see Bohne et al., 2016). Typically, a TPR domain consists of multiple copies (3–16) of a degenerate motif which comprises 34 amino acids forming two amphipathic α-helices. The crystal structure of TPR domains revealed that these form right-handed superhelices that serve as a platform for protein–protein interactions (Blatch and Lässle, 1999; D’Andrea and Regan, 2003). TPR proteins have been implicated in a variety of functions during the biogenesis of TMs, including chloroplast protein import, gene expression and chlorophyll (Chl) synthesis, as well as PSII and PSI assembly (Bohne et al., 2016). In total, the *Synechocystis* genome encodes 29 TPR proteins (Bohne et al., 2016). These include Ycf3 and Ycf37, which have been shown to facilitate PSI assembly. The TPR protein Pitt (light-dependent protochlorophyllide oxidoreductase interacting TPR protein) interacts with POR (light-dependent protochlorophyllide oxidoreductase) and regulates Chl synthesis (Schottkowski et al., 2009b; Rengstl et al., 2011). For cyanobacterial PSII assembly, the above-mentioned TPR protein PratA plays an important role and recently the protein Slr0151 has been shown to be involved in the PSII repair cycle (Yang et al., 2014).

The *slr0151* gene is part of the *slr0144-slr0151* operon, which codes for eight proteins (Kopf et al., 2014; Yang et al., 2014). This operon was first discovered in the course of a microarray analysis in which expression of the cluster was down-regulated under iron-depleted conditions and during oxidative stress (Singh et al., 2004). The authors hypothesized that the gene cluster is involved in PSI assembly (Singh et al., 2004), and indeed a second study found Slr0151 to be associated with PSI complexes (Kubota et al., 2010). Others, however, have pointed to connections between Slr0151 and PSII (Wegener et al., 2008; Yang et al., 2014). Thus, Wegener et al. (2008) referred to the proteins encoded by the *slr0144-slr0151* operon as PSII assembly proteins (Pap) because they stabilize PSII intermediates. Furthermore, these authors showed that the entire *pap* operon is up-regulated upon loss of any of the lumenal proteins CyanoP, PsbV, and CyanoQ (Wegener et al., 2008). In a *slr0151*^-^ mutant, however, the expression of the other *pap* genes was not affected (Yang et al., 2014). In addition, experimental evidence has been provided that links Slr0151 to the PSII repair cycle, and yeast two-hybrid and pulldown analyses have revealed that Slr0151 interacts directly with both CP43 and D1 (Yang et al., 2014). In this study, we further characterize the function and subcellular localization of Slr0151.

## Materials and Methods

### Construction and Growth of Strains

*Synechocystis* wild-type and mutant strains were grown on solid or in liquid BG-11 medium at 30°C at a continuous photon irradiance of 30 μmol photons m^-2^ s^-1^. The insertion mutant *slr0151*^-^ was generated by PCR amplification of the wild-type *slr0151* gene with the oligonucleotides 0151/5 ATGATGGAAAATCAAGTTAATGA and 0151/3 TTAACCAAATAGGTTAGCTGC as primers, and subsequent cloning of the resulting fragment into the pDrive vector (Qiagen). The fragment was cut from pDrive and inserted into Bluescript pKS vector via the restriction enzymes SalI and PstI of both multiple cloning sites. A kanamycin-resistance cassette was then inserted into its unique HindIII restriction site, and wild-type cells were transformed with the construct as described. For complementation of the *slr0151*^-^ mutant, the *slr0151* gene (including its own promoter) was PCR-amplified with oligonucleotides 0151/5b CTCGAGTGATGAGTTTTTTTAGCTCTA and 0151/3b CTCGAGAACTGGAGTTTTAACCAAA, and cloned into the single XhoI site in the vector pVZ321, which replicates autonomously in *Synechocystis* 6803 (Zinchenko et al., 1999). Transfer of this construct into *slr0151*^-^ via conjugation was performed as described (Zinchenko et al., 1999). Construction of the mutant lines *psbA*^-^ (*TD41*), (Nixon et al., 1992), *ctpA*^-^ (Rengstl et al., 2011), *pratA*^-^ (Klinkert et al., 2004), *psbB*^-^ (Eaton-Rye and Vermaas, 1991), *ycf48*^-^ (Komenda et al., 2008), *sll0933*^-^ (Armbruster et al., 2010), *psb27*^-^ (Komenda et al., 2012), and *pitt*^-^ (Schottkowski et al., 2009b) was described previously.

### Antibody Production and Western Analysis

For production of the αSlr0151 antibody, the *slr0151* reading frame without the N-terminal transmembrane region (amino acid positions 62 to 320) was PCR-amplified using oligonucleotides TH0151a GGATCCGAATTCCATTTGTTTAACCGTAAGCAGTT and TH0151b GTCGACTTAACCAAATAGGTTAGCTGCGGT. The resulting DNA fragment was inserted into the pDrive vector (Qiagen), sequenced and further subcloned into the BamHI and SalI restriction sites of the expression vector pGex-4T-1. Expression of the GST fusion protein in *Escherichia coli* BL21 and its affinity purification on Glutathione-Sepharose 4B (GE Healthcare) were performed according to the manufacturers’ instructions. Polyclonal antiserum was raised in rabbits (Biogenes). Protein preparation from *Synechocystis* 6803 and western analyses were carried out as previously reported (Wilde et al., 2001).

### Isolation of Total Cell Protein, Membrane Fractionation, and Western Analysis

Isolation of whole-cell protein, two-step membrane fractionation via consecutive sucrose-density gradients, and western analyses were carried out as described previously (Wilde et al., 2001; Schottkowski et al., 2009a,b; Rengstl et al., 2011). Apart from αSlr0151, the primary antibodies used in this study have been described earlier, i.e., αD1 (Schottkowski et al., 2009b), αD2 (Klinkert et al., 2004), αPratA (Klinkert et al., 2004), αYcf48 (Rengstl et al., 2011), αPitt (Schottkowski et al., 2009a), αPOR (Schottkowski et al., 2009a), αYidC (Ossenbühl et al., 2006), αSll0933 (Armbruster et al., 2010), or were purchased from Agrisera (Vännäs, Sweden), i.e., αCP43, αCP47, αRbcL. Western blots were quantified using AIDA software (version 3.52.046). For each experiment, the respective RbcL signal served as internal loading control. Quantifications are based on at least three independent experiments. Mean and standard deviation were calculated for the protein levels and Student’s *t*-test was performed to verify statistical significant differences between wild-type and *slr0151*^-^.

### Transmission Electron Microscopy

*Synechocystis* cells (*slr0151*^-^ mutant and wild-type) were harvested in mid-log phase by centrifugation at 5000 *g* and adjusted to an OD_750__nm_ = 3 in BG-11. Aliquots (2 μl) of the cell suspension were high-pressure frozen at 2100 bar (Leica HPM 100) in HPF gold platelets (Leica Microsystems, Vienna, Austria) and stored in liquid nitrogen (Rachel et al., 2010; Klingl et al., 2011). The cryofixed cells were then freeze-substituted (Leica EM AFS2) at -90°C with 2% osmium tetroxide and 0.2% uranyl acetate in pure acetone. Freeze substitution was carried out at -90°C for 20 h, -60°C for 8 h, -30°C for 8 h, with a heating time of 1 h between each step, and then held at 0°C for 3 h. Samples were washed three times with pure, ice-cold acetone followed by infiltration with Epon resin (Fluka, Buchs, Switzerland). After polymerization for 72 h at 63°C, ultrathin sections were cut, and post-stained with lead citrate (Reynolds, 1963). Transmission electron microscopy was carried out at 80 kV either on a Zeiss EM 912 or on a Fei Morgagni 268 electron microscope (FEI). Data analysis was carried out with the Fiji ImageJ software.

### Immunofluorescence and Fluorescence Microscopy

*Synechocystis* cells were harvested in mid-log phase by centrifugation at 5000 *g* and adjusted to an OD_750__nm_ = 3 in PBS (140 mM NaCl, 2.7 mM KCl, 10 mM Na_2_HPO_4_, 1.8 mM KH_2_PO_4_; pH 7.4). The cells were fixed with 2% formaldehyde (35%, for Histology, Roth) in PBS for 20 min at 30°C on a shaker, then washed twice with PBS-T (PBS supplemented with 0.05% Tween-20). For permeabilization, the cells were incubated with PBS-T for 2 min × 3 min on an overhead rotor. All subsequent steps were performed in the dark. The cells were applied to poly-L-lysine-coated glass slides (Sigma) and incubated for 30 min to allow them to settle, then incubated with blocking buffer (5% non-fat milk powder in PBS-T) for 20 min. The slides were incubated for 3 h with the first antibody (αSlr0151 and αRbcL, diluted 1:500 in blocking buffer, then washed for 3 min × 3 min by incubating them with PBS-T and rinsing the solution off the slide. The secondary HRP-conjugated goat anti-rabbit antibody (Sigma) was labeled with Alexa 488 (Alexa Fluor Dyes, Life Technologies, ThermoFisher Scientific) according the manufacturer’s instruction. The slides were incubated with the labeled secondary antibody (diluted 1:2000 in blocking buffer) for 1 h. The slides were washed twice with PBS-T and twice with PBS-G (PBS supplemented with 10 mM glycine) to minimize background fluorescence due to non-labeled fluorophores. The slides were then dried and each was covered with a drop of FluorSave^TM^ Reagent (Calbiochem, Merck Millipore) and a coverslip. Next day, the coverslip was sealed with nail polish. Fluorescence was imaged using a Delta Vision Elite (GE Healthcare, Applied Precision) equipped with Insight SSI^TM^ illumination and a CoolSNAP_HQ2 CCD camera. Cells were imaged with a 100× oil PSF U-Plan S-Apo 1.4 objective. The four-color standard set InsightSSI module (code number: 52-852113-003, GE Healthcare, Applied Precision) was used for imaging. Alexa488 was excited with the FITC/GFP excitation filter (461–489 nm) and emission was detected with the FITC/GFP emission filter (501–549 nm). Chlorophyll autofluorescence was excited with the TRITC/Cy3-filter (528–555 nm) and emission was detected with the TRITC/Cy3 filter (574–619 nm). Images were analyzed using the Fiji ImageJ software.

## Results

### Molecular *slr0151*^-^ Phenotype

The *slr0151* open reading frame in *Synechocystis* encodes a protein of 320 amino acids, which contains two consecutive TPR domains comprising positions 185–218 and 219–252 (analyzed with TPRpred; Yang et al., 2014; Bohne et al., 2016). It has previously been shown that Slr0151 is an intrinsic membrane protein which forms part of a high-molecular-weight complex (Yang et al., 2014).

To analyze the function of Slr0151, we disrupted its cloned reading frame by inserting a kanamycin-resistance cassette into the unique HindIII site 425 bp downstream of the start codon (**Supplementary Figure S1A**). After transformation of wild-type (WT) cells with this construct, the transformants were tested for complete segregation by PCR analysis (**Supplementary Figure S1B**). The complete absence of the Slr0151 protein in the *slr0151*^-^ mutant was verified by western analysis using an αSlr0151 antibody (**Supplementary Figure S1C**). Like the previously described *slr0151*^-^ mutant, the mutant strain described in this study exhibited a high light (800 μmol photons m^-2^ s^-1^) sensitive phenotype (Yang et al., 2014). Moreover, and also in agreement with the previous report, less pronounced effects were observed under normal lighting conditions (30 μmol photons m^-2^ s^-1^). These effects included moderately reduced photoautotrophic growth and oxygen production rates (Yang et al., 2014). Together, these findings suggest that, like other PSII assembly factors such as Ycf48 or Psb27, Slr0151 might be involved in both PSII assembly and repair (Komenda et al., 2012; Rengstl et al., 2013; Mabbitt et al., 2014; Jackson and Eaton-Rye, 2015).

**FIGURE 1:**
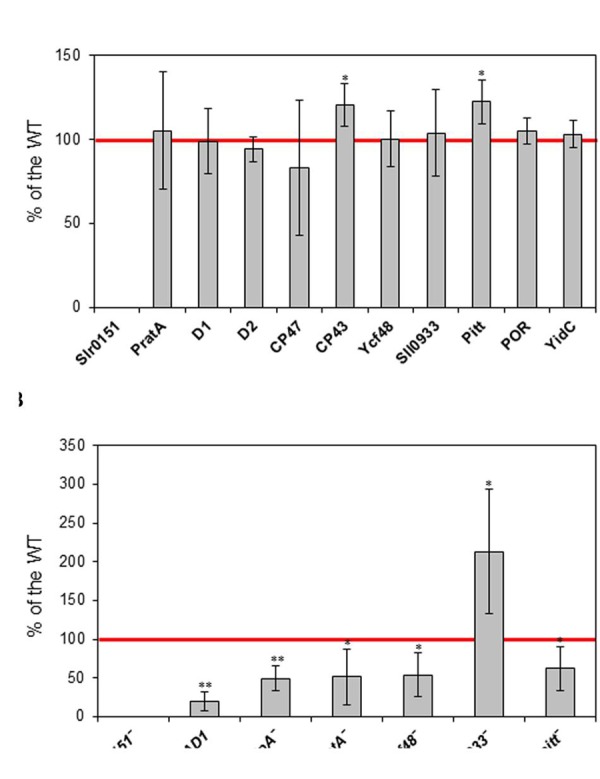
**Steady-state levels of the indicated PSII-related proteins in the *slr0151*^-^ mutant **(A)** and of Slr0151 in the indicated PSII mutants **(B)**.** Each value is expressed relative to that in the wild type. Total cell proteins were isolated, fractionated by SDS-PAGE, blotted onto nitrocellulose membranes and detected immunologically. Signals were quantified with AIDA software (version 3.52.046) after densitometrical scanning. Values plotted are means ± SD of at least three independent experiments. Significance according to Student’s *t*-test with an error probability of 5 and 1% is indicated by one and two asterisks, respectively.

To explore this possibility further, the levels of various photosynthetic subunits and PSII biogenesis factors accumulated in the *slr0151*^-^ strain under normal growth conditions were analyzed. Almost all analyzed proteins showed no significant differences relative to WT, except CP43 and Pitt had increased levels in the mutant (**Figure 1A**; **Supplementary Figure S2A**). Conversely, however, levels of Slr0151 were significantly altered in different PSII assembly mutants (**Figure 1B**; **Supplementary Figure S2B**). In the Δ*D1* mutant, in which all three copies of the *psbA* gene are inactivated, Slr0151 was reduced to only 20% of its wild-type level (Nixon et al., 1992). The *ctpA*^-^ mutant lacking the C-terminal processing protease for pD1 accumulated only 50% as much Slr0151 as did wild-type cells (Anbudurai et al., 1994). In the PSII assembly factor mutants *pratA*^-^, *ycf48*^-^, and *pitt*^-^ (Klinkert et al., 2004; Komenda et al., 2008; Schottkowski et al., 2009b) amounts of Slr0151 reached 52, 53, and 62% of the wild-type level, respectively. In sharp contrast, however, more than twice the WT level of Slr0151 was detected in *sll0933*^-^, which lacks the cyanobacterial homolog of the *Arabidopsis* PSII assembly factor PAM68. This suggests a functional relationship between Slr0151 and Sll0933, despite the fact that levels of the latter were unchanged in the *slr0151*^-^ mutant (**Figure 1**; **Supplementary Figure S2**). To test if this relationship relies on a physical interaction of both factors, we performed co-immunoprecipitation experiments. However, no interaction between Slr0151 and Sll0933 were detected under the applied conditions suggesting that they do not form parts of a stable complex *in vivo*.

### Ultrastructure of *slr0151*^-^ Cells

**FIGURE 2:**
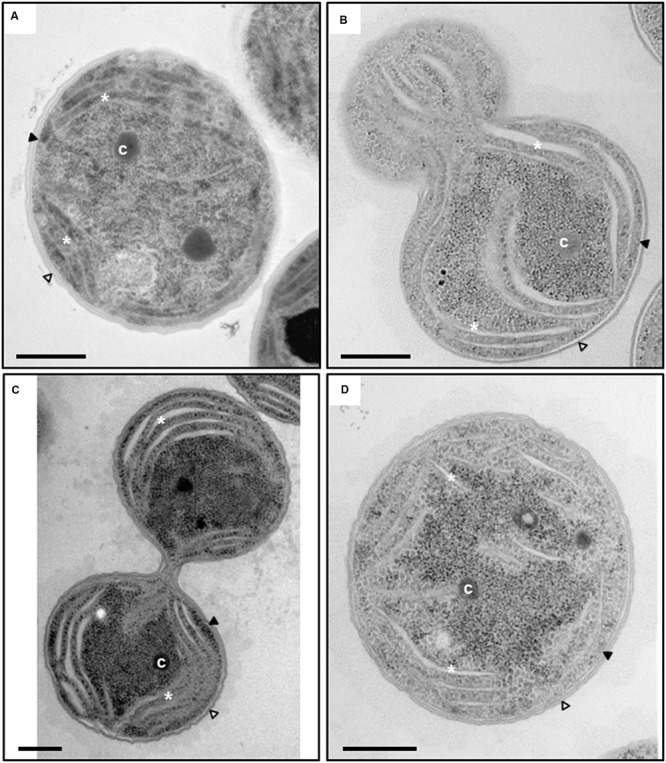
**Transmission electron microscopy of wild-type *Synechocystis***(A)** and *slr0151*^-^ mutant cells **(B–D)**.** Different stages during the cell division of the *slr0151*^-^ strain are shown; early stage of the cell division **(B)**, late stage of cell division right before cytokinesis **(C)** and non-dividing single cell **(D)**. Intracellular components are labeled: Outer membrane (white arrowhead), plasma membrane (black arrowhead), thylakoids (white asterisks) and carboxysome (**c**). Bars = 500 nm.

In order to gain more insight into the subcellular consequences of *slr0151* inactivation, the ultrastructure of the mutant was visualized by transmission electron microscopy (**Figure 2**). In *slr0151*^-^ cells grown at normal light intensities, the thylakoids appeared to be less densely packed and thylakoid lumina were swollen when compared to the wild-type (**Figure 2**). Lumen diameters ranged from 4 to 91 nm in the mutant, whereas the values for wild-type thylakoids fell within the 5- to 9-nm range, as previously observed (**Figure 2**; van de Meene et al., 2006). Thus, in addition to PSII assembly/repair, Slr0151 deficiency appears to affect TM organization.

### Localization of Slr0151 and PSII-Related Factors in Membrane Subfractions

**FIGURE 3:**
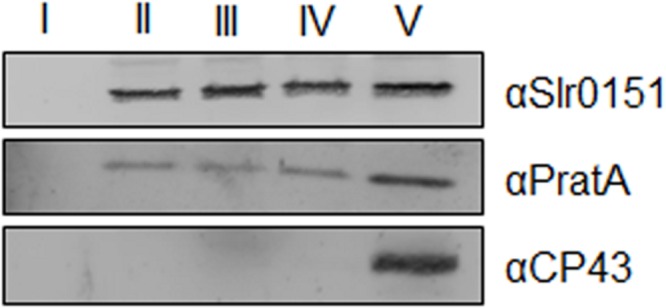
**Membrane localization of Slr0151.** Sucrose step-gradient centrifugation was used to separate membrane types from wild-type *Synechocystis* cells. Fraction II corresponds to the plasma membrane (PM) and fraction V contains pratA-defined biogenic membranes (PDMs) + thylakoid membrane (TMs). For fractions I–IV 10% of the total volume of each fraction was loaded, while only 0.2% of fraction V was analyzed (Schottkowski et al., 2009a).

**FIGURE 4:**
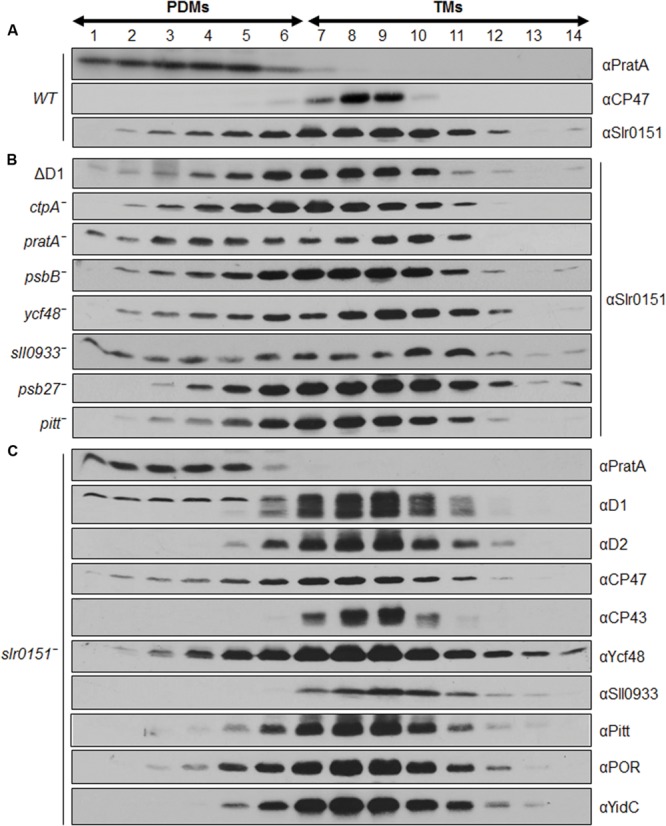
**Distribution of Slr0151 between PDMs and TMs. (A)** Wild-type fraction V (see **Figure 3**) was centrifuged on linear sucrose gradients in order to separate wild-type PDMs from TMs. **(B)** Distribution of Slr0151 between PDMs and TMs in the indicated PSII mutants. **(C)** Distribution of various PSII-related proteins in *slr0151*^-^ cells. The part of the gradient from 20 to 60% sucrose was apportioned into 14 fractions, which were analyzed by immunoblotting using the antibodies indicated on the right. Fractions 1–6 represent PDMs, and fractions 7–14 represent TMs. To facilitate comparison between gradients, sample volumes were normalized to the volume of fraction 7 that contained 40 μg of protein.

Slr0151 has previously been reported to localize to both the PM and TMs, based on a combined sucrose density/two-phase partitioning approach (Yang et al., 2014). Alternatively, cyanobacterial membranes can be fractionated into PM and TMs via a sucrose step gradient, and the latter can be further fractionated into PratA-defined biogenic membranes (PDMs) and photosynthetically active thylakoids on a second, linear sucrose gradient (Schottkowski et al., 2009a; Heinz et al., 2016). When the distribution of Slr0151 in membrane sub-fractions was followed by applying the latter technique, the protein was accordingly detected in both PM and TM fractions (**Figure 3**). Further fractionation of thylakoids then revealed that Slr0151 is found in both PDMs and TMs, indicating that the protein is broadly distributed throughout the cell (**Figure 4A**). Since Slr0151 accumulation was affected in several PSII-related mutants, we next analyzed its TM distribution in the various mutant backgrounds (**Figures 2B** and **4B**). In most cases, Slr0151 distribution followed the wild-type pattern. The only exceptions were the *pratA*^-^ and *sll0933*^-^ mutants (**Figures 4A,B**). In these strains, a shift of Slr0151-containing material toward the less dense PDM fractions was observed (**Figures 4A,B**). This again suggested a functional relationship between Slr0151 and the PAM68 homolog Sll0933 and, furthermore, a connection to the biogenic PratA-defined region at the periphery of the cell.

When the distribution of several PSII-related proteins was monitored in a *slr0151*^-^ background, no significant effects were seen (**Figure 4C**; Rengstl et al., 2011). The only alteration in membrane distribution concerned CP47. This is usually seen exclusively in TMs, but accumulates to some extent in PDM fractions in the absence of Slr0151 (**Figures 4A,C**). However, Slr0151 localization was not affected in a *psbB*^-^ mutant (**Figure 4B**). Taken together, these data revealed a broad membrane distribution of Slr0151 and further confirmed its relationship to PSII assembly/repair. The distribution Slr0151 in the WT is almost identical to the distribution of Ycf48 which has been shown to be involved in assembly and repair of PSII.

### Localization of Slr0151 via Immunofluorescence

**FIGURE 5:**
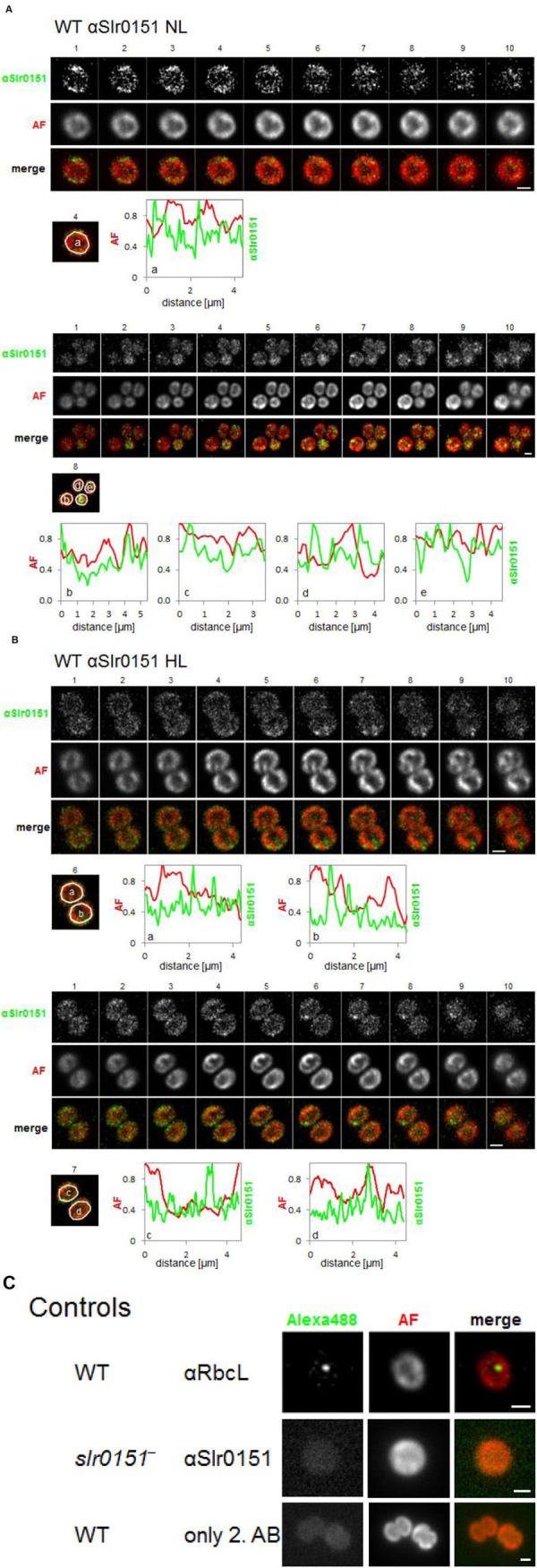
**Subcellular localization of Slr0151 by immunofluorescence analysis of wild-type *Synechocystis* cells.** Cells grown under normal light conditions **(A)** and cells that had been exposed to high light for 1 h **(B)** were treated with αSlr0151 antibody and detected with an Alexa-488-coupled secondary antibody. The constituent subpanels show Z-montages (200 nm spacing) of each separate channel as well as the merged channel. The line plots of relative fluorescence signals are derived from scans of the area defined by the white circle around the indicated cells, and correspond to the *Z*-slice number of the analyzed slice (given at the top). **(C)** For controls, cells were grown under normal conditions. In the upper row, *Synechocystis* wild-type cells were immunostained with αRbcL and visualized with Alexa-488 coupled secondary antibody. In the middle row, *slr0151*^-^ mutant cells have been treated with the αSlr0151 antibody followed by Alexa-488-coupled secondary antibody. In the bottom row, wild-type cells were probed with Alexa-488-coupled secondary antibody alone. AF, autofluorescence of chlorophyll; scale bar 1 μm.

To obtain a more comprehensive view of the subcellular localization of Slr0151, we next performed immunofluorescence (IF) analyses with affinity-purified αSlr0151, in combination with an Alexa488-labeled secondary antibody. Fluorescence was recorded by wide-field microscopy followed by deconvolution. In **Figure 5**, Z-montages from different cells are shown that display sequential slices from the Z-stack to provide a better 3D representation of whole cell volumes. Overall, Slr0151 IF signals in wild-type cells grown under normal light conditions were unevenly distributed, with frequent spot-like concentrations. These partly coincided with the Chl autofluorescence of the thylakoids, but were also detected in regions with low Chl fluorescence, i.e., in the PM at the cell periphery and in thylakoid convergence zones close to the PM. Interestingly, fluorescence signals were also visible in Chl-less central regions of the cells, where fewer thylakoid lamellae tend to traverse the cytoplasm (**Figure 5A**). Thus, the fluorescence signal is in accordance with the observations from membrane fraction analysis, i.e., that Slr0151 is located in the PM, and in PDMs and TMs. In addition, these data provide evidence that Slr0151 is found in punctate concentrations within the membrane, reminiscent of the previously described distribution of FtsH2-GFP signals, which are thought to label PSII repair zones (Sacharz et al., 2015). Similar to FtsH-GFP signals, Slr0151 IF patterns were unchanged after a 1-h exposure to high light, suggesting that an enhanced requirement for PSII repair does not provoke any substantial reorganization of Slr0151 localization (**Figure 5B**; Sacharz et al., 2015).

Control experiments included the omission of the specific αSlr0151 antibody, and analysis of the *slr0151*^-^ mutant, which displayed at most diffuse background signals (**Figure 5C**). Moreover, we used an αRbcL antibody as a control for a non-membrane protein that gives rise to fluorescence labeling of carboxysomes from *Synechocystis* (Cameron et al., 2013).

## Discussion

Yang et al. (2014) demonstrated that the TPR protein Slr0151 is involved in the repair of PSII in *Synechocystis* cultures grown at high light. Prompted by the finding that a milder phenotype can be observed under normal lighting conditions, we have carried out a further investigation of the effects of loss of Slr0151 in that context. In particular, our data suggest a functional relationship between Slr0151 and the PSII assembly factor Sll0933, a homolog of the PAM68 protein from *A. thaliana*, which has been shown to play a role in the conversion of RC complexes into larger PSII pre-complexes by facilitating attachment of the inner antenna proteins (**Figure 1B**; Armbruster et al., 2010; Rengstl et al., 2013).

In agreement with the idea that Slr0151 is involved in the transition to larger PSII pre-complexes, reduced amounts of the RC47 complex have previously been detected in *slr0151*^-^ cells grown under high light (Yang et al., 2014). Moreover, the direct interaction of Slr0151 with CP43 and D1, as well as the unusual membrane distribution of CP47 in PDMs in the *slr0151*^-^ background, suggest a role for Slr0151 in the transition from RC complexes to PSII monomers (**Figure 4C**; Yang et al., 2014). Interestingly, PDM-localized CP47 fractions have also been observed in a *ctpA*^-^ mutant, which further supports the idea that Slr0151 acts during the transition from the RC47 complex to the PSII monomer lacking the OEC (Rengstl et al., 2011). Ycf48 is involved in the formation of the RC complex during assembly and repair of PSII (Komenda et al., 2008; Rengstl et al., 2011). Interestingly, it is distributed like Slr0151 in membrane fractionation experiments (**Figure 4**). This might suggest that a broad membrane distribution of PSII related proteins is characteristic for factors being involved in both PSII assembly and repair. Taken together, these findings confirm a PSII-related function for Slr0151, and demonstrate that, even under normal lighting conditions, distinct molecular phenotypes are detectable upon inactivation of Slr0151.

This is further underlined by the altered ultrastructure of thylakoids, i.e., looser membrane packing and increased lumen volume, seen in the *slr0151*^-^ mutant grown in normal light. Swollen lumina of thylakoids have been observed before in WT cells grown at 0.5 μmol photons m^-2^ s^-1^ and in WT and mutants with different depletions of carotenoids grown in the dark with 10 min of light per day (Van de Meene et al., 2012; Toth et al., 2015). Recently, a study using inelastic neutron scattering on living *Synechocystis* WT cells investigated the membrane dynamics of thylakoids during light and dark periods (Stingaciu et al., 2016). The authors showed that the TM in *Synechocystis* is less flexible in the light as compared to dark conditions due to formation of the photosynthetic proton gradient across the TMs. Therefore, it appears possible that distorted photosynthesis or an absence of the structural function of Slr0151 itself causes swollen thylakoids in the *slr0151^-^* mutant. Such a structural role would also be in line with the observed broader membrane distribution of Slr0151. Taken together, we propose that Slr0151 – like other PSII assembly factors such as CtpA, Ycf48 and Psb27 – is involved in both PSII assembly and repair (Nowaczyk et al., 2006; Komenda et al., 2007; Nickelsen and Rengstl, 2013; Jackson et al., 2014; Mabbitt et al., 2014). The suggested involvement of Slr0151 in both processes is also consistent with the fact that the RC47 complex represents the point of convergence between them.

Slr0151 is an intrinsic membrane protein that does not accumulate in the cytoplasm (Yang et al., 2014; data not shown). Indeed, it can be found in a variety of specialized membrane domains. Previously, Slr0151 was detected in the PM as well as in the thylakoids of *Synechocystis* (Huang et al., 2002; Yang et al., 2014). This distribution was confirmed by our membrane fraction experiments (**Figures 3** and **4**). In addition, substantial amounts of Slr0151 were observed in PDMs, which are localized at sites where thylakoids converge upon the PM. According to rough estimates based on densitometrical signal analysis, approximately 2% of total cellular Slr0151 is found in PMs and 25 and 70% in PDMs and TMs, respectively. IF analyses confirmed this overall distribution and revealed frequent punctate concentrations of Slr0151 in all membrane types (**Figure 5**). Intriguingly, a similar localization pattern has been observed for GFP-tagged FtsH2, the protease which degrades damaged D1 protein during PSII repair. The GFP signal co-localized with the Chl autofluorescence and showed patches of increased intensity within as well as between thylakoids at their peripheral convergence sites (Sacharz et al., 2015). The same patterns were maintained under high light conditions for both Slr0151 and FtsH2 (Sacharz et al., 2015). Thus, these data, together with the observation that the synthesis of D1 following photodamage is affected by Slr0151 inactivation (Yang et al., 2014), are consistent with a role of Slr0151 during repair. Furthermore, IF analysis has shown that some Slr0151 is concentrated in thylakoids that traverse the cell center. Whether this reflects any distinct functional role of these regions remains to be discovered.

This work reveals new aspects of the function of the TPR protein Slr0151, i.e., its involvement in PSII assembly in addition to its previously described role in PSII repair. The fact that PSII assembly takes place in BCs does not exclude the possibility that repair and assembly of PSII are co-localized in those regions. Since several steps of assembly and repair involve the same assembly/repair factors as well as some assembly and assembly repair intermediates (Schottkowski et al., 2009a; Rengstl et al., 2011; Stengel et al., 2012). Therefore, the current findings suggest a close relationship between PSII assembly and repair, with regard to the factors involved and their subcellular distribution (Nickelsen and Rengstl, 2013; Mabbitt et al., 2014).

## Author Contributions

AR, BR, SH, AK, and JN designed the research. AR, BR, and SH performed the research. AR and JN perpared the article.

## Conflict of Interest Statement

The authors declare that the research was conducted in the absence of any commercial or financial relationships that could be construed as a potential conflict of interest.

The reviewer SJ and handling Editor declared their shared affiliation, and the handling Editor states that the process nevertheless met the standards of a fair and objective review.

## Supplementary Material

FIGURE S1 **Generation of the *Synechocystis slr0151*^-^ mutant. (A)** Construction of the insertional *slr0151*^-^ mutant. **(B)** PCR-based segregation analysis of the *Synechocystis slr0151*^-^ mutant [primer locations are indicated by black arrows in **(A)**]. **(C)** Levels of Slr0151 protein detected with αSlr0151 antiserum in the wild-type (WT), *slr0151*^-^ mutant and the complemented *slr0151*^-^ strain (*re-slr0151*^-^). RbcL served as the loading control.

FIGURE S2 **Protein levels in the *slr0151*^-^ mutant and of Slr0151 in various PSII mutants.** Representative western blots from the protein level analysis shown in **Figure 1**. Total proteins were isolated from the respective line and analyzed via SDS-PAGE and western blot. 30 μg were loaded for 100% wild type and each mutant. The quantification of at least three independent experiments is summarized in **Figure 1**. The RbcL signal served as internal standard for relative quantification. **(A)** Protein levels of the indicated PSII subunits and PSII-related proteins in the *slr0151*^-^ mutant. Wild-type and mutant samples were analyzed on the same gel. However, signals from unrelated samples, which were loaded in between, were excised. **(B)** Representative western analysis of Slr0151 in various PSII mutants.
