# Supplementary figures and images for: The Role of Slr0151, a Tetratricopeptide Repeat Protein from Synechocystis sp. PCC 6803, during Photosystem II Assembly and Repair
Source: Front Plant Sci. 2016 May 3;7:605. doi: 10.3389/fpls.2016.00605 (PMC4853703; doi:10.3389/fpls.2016.00605)

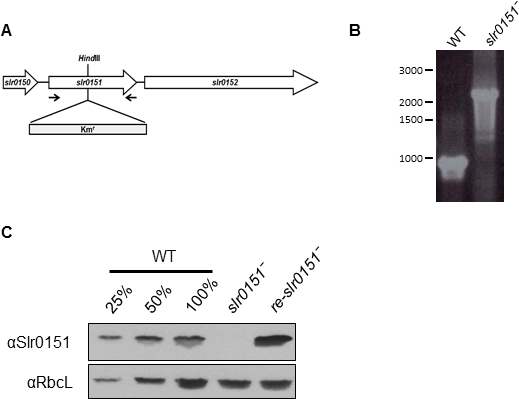

Supplement: FIGURE S1 — Generation of the Synechocystis slr0151- mutant. (A) Construction of the insertional slr0151- mutant. (B) PCR-based segregation analysis of the Synechocystis slr0151- mutant [primer locations are indicated by black arrows in (A)]. (C) Levels of Slr0151 protein detected with αSlr0151 antiserum in the wild-type (WT), slr0151- mutant and the complemented slr0151- strain (re-slr0151-). RbcL served as the loading control. [file Image_1.TIF]

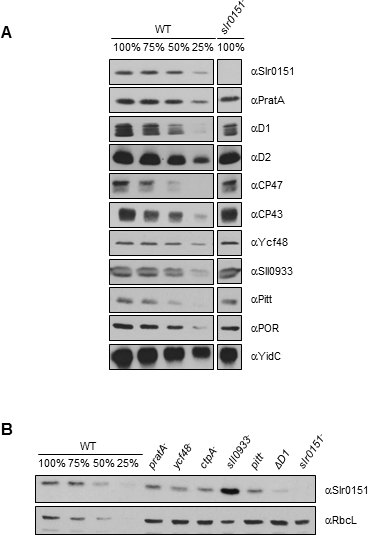

Supplement: FIGURE S2 — Protein levels in the slr0151- mutant and of Slr0151 in various PSII mutants. Representative western blots from the protein level analysis shown in Figure 1. Total proteins were isolated from the respective line and analyzed via SDS-PAGE and western blot. 30 μg were loaded for 100% wild type and each mutant. The quantification of at least three independent experiments is summarized in Figure 1. The RbcL signal served as internal standard for relative quantification. (A) Protein levels of the indicated PSII subunits and PSII-related proteins in the slr0151- mutant. Wild-type and mutant samples were analyzed on the same gel. However, signals from unrelated samples, which were loaded in between, were excised. (B) Representative western analysis of Slr0151 in various PSII mutants. [file Image_2.TIF]
